# Impact of *Suaahara*, an integrated nutrition programme, on maternal and child nutrition at scale in Nepal

**DOI:** 10.1111/mcn.13630

**Published:** 2024-02-11

**Authors:** Edward A. Frongillo, Shalini Suresh, Deependra K. Thapa, Kenda Cunningham, Pooja Pandey Rana, Ramesh P. Adhikari, Subir Kole, Bhim Pun, Indra Kshetri, Debendra P. Adhikari, Rolf Klemm

**Affiliations:** ^1^ Department of Health Promotion, Education, and Behavior, Arnold School of Public Health University of South Carolina Columbia South Carolina USA; ^2^ Independent Consultant Stamford Connecticut USA; ^3^ Nepal Public Health Research and Development Center Kathmandu Nepal; ^4^ Helen Keller International New York City New York USA; ^5^ United States Agency for International Development Kathmandu Nepal

**Keywords:** behaviour, breast feeding, child, diet, mothers, Nepal, nutritional status

## Abstract

*Suaahara* was an innovative, complex, multi‐sectoral, large‐scale, nutrition programme in Nepal to increase exposure to nutrition‐related information and services, improve nutrition‐related knowledge and practices among pregnant women and mothers of infants and young children, and improve their nutrition. This study evaluated the effectiveness of *Suaahara* to improve nutrition and nutrition‐related practices by comparing changes over 10 years between intervention and comparison districts. The samples of households at baseline in 2012 and endline in 2022 were 2040 and 2480, respectively, from 120 old wards. The impact was estimated using intent‐to‐treat regression models in which survey year, arm and their interaction were fixed effects, accounting for district clustering, with the interaction estimating differences between arms in changes over time. The intervention, relative to comparison, reduced maternal underweight by 8.43 percentage points (*p* < 0.001), consistent with improved maternal and fetal condition that was manifested as the greater length of 0.761 *z*‐scores (*p* = 0.004) of infants 0–5.9 months. Complementary feeding practices with children between 6 and 23.9 months of age improved more in the intervention than comparison districts: child dietary diversity by 0.294 food groups (*p* = 0.072) and minimum dietary diversity by 9.51 percentage points (*p* = 0.028), feeding sick child more (*p* = 0.002) and administering oral rehydration solution and zinc for diarrhoea (*p* = 0.057) by about 17 percentage points each, and minimum meal frequency (*p* = 0.004) and minimum acceptable diet (*p* = 0.022) by about 15 percentage points each. Substantial impacts were demonstrated despite political restructuring, earthquakes, and other major challenges that Nepal and *Suaahara* faced and limitations in statistical power because of the reduced number of districts that then could be included in the study. Registered at clinicaltrials.gov with identifier NCT05448287.

## Key messages

- *Suaahara* reduced maternal underweight, consistent with improved maternal and fetal condition that was manifested in the greater length of 0–5.9 months old infants.
- *Suaahara* improved complementary feeding practices with children ages 6–23.9 months: child dietary diversity, minimum meal frequency, minimum acceptable diet, feeding sick children more and administering oral rehydration solution and zinc for diarrhoea.
- Substantial improvements in the nutrition of mothers and young children from *Suaahara* demonstrate that a multi‐sectoral, large‐scale, nutrition programme in Nepal can reduce undernutrition.

## Graphical abstract

*Suaahara*, relative to comparison areas, reduced maternal underweight and improved complementary feeding practices with children 6–23.9 months of age, increasing the percentages of children having minimum dietary diversity, minimum meal frequency and minimum acceptable diet; feeding sick child more and administering oral rehydration solution and zinc for diarrhoea.

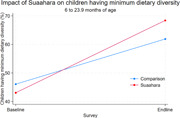

## 1 INTRODUCTION

Undernutrition of mothers and children remains prevalent in South Asia (Black et al., 2013; Shekar et al., 2016), with consequences for the health of mothers and the physical and psychomotor development of children (Hoddinott et al., 2011; Hoddinott, Alderman, et al., 2013; Hoddinott, Behrman, et al., 2013). Nutrition‐specific and ‐sensitive actions to reduce undernutrition have concentrated on mothers and young children during their first 1000 days between conception and 24 months of age (Horton & Lo, 2013). Nutrition‐specific actions address nutrition directly through improving food intake and preventing disease, primarily delivered through health or community systems. Nutrition‐sensitive actions address underlying determinants that are linked to nutrition, that is, household food insecurity, inadequate care and lack of access to health services and a healthy environment (UNICEF, 1990). These actions may occur in several sectors such as education, agriculture and water, sanitation and hygiene (WASH), and may have both direct and indirect links with nutrition. For example, nutrition‐sensitive agriculture interventions positively impact child dietary diversity (Margolies et al., 2022). Knowledge of how to design and implement complex, integrated actions in multiple sectors at scale, however, is nascent (Bhutta et al., 2008; Menon et al., 2014; Ruel & Alderman, 2013).

Although Nepal reduced maternal and child undernutrition over the past 30 years, undernutrition persists. In 2012, 41% of children under 5 years of age were stunted and 11% were wasted (Ministry of Health and Population [MoHP] Nepal, 2012). Improvements in Nepal in education and WASH have contributed to the reduction in undernutrition (Crum et al., 2013; Cunningham et al., 2016; Headey & Hoddinott, 2015; Headey et al., 2016), although reductions are uneven. Other possible reasons for improvement are wider coverage of nutrition and health services, vitamin A supplementation, improvement of household wealth and implementation of a multi‐sectoral nutritional plan (Chitekwe et al., 2022). When undernutrition is indicated by child stunting, the prevalence is higher in rural than urban areas, in those with less (vs. greater) wealth and in those with historically disadvantaged caste groups (MoHP Nepal, 2012). Unequal access to resources and services, political representation and opportunities have resulted from geographic isolation and long‐term social and economic inequities (Anon, 2006; Crum et al., 2013; Devkota & Bennett, 2014; Devkota et al., 2016).

*Suaahara* was initiated in 2011 as an innovative, complex, multi‐sectoral nutrition programme in Nepal designed to be implemented at a large scale. *Suaahara* intended to increase exposure to nutrition‐related information and services, thereby improving nutrition‐related knowledge and practices among pregnant women and mothers of infants and young children in Nepal and ultimately improving their nutrition. *Suaahara* intended to narrow equity gaps in nutrition‐related exposures, knowledge and practices, in part by targeting efforts in remote communities and for marginalized populations. This study aimed to evaluate the effectiveness of *Suaahara* to improve nutrition and nutrition‐related practices by comparing changes over 10 years between intervention districts that participated in the *Suaahara* programme and comparison districts that did not and to explore whether inequity was reduced.

## 2 METHODS

### 2.1 *Suaahara* programme

*Suaahara* was funded by the United States Agency for International Development and implemented in two phases (Phase I: 2012–2016 and Phase II: 2017‐2021, extended to 2023) as an integrated programme to improve the nutrition of women and children in 42 of Nepal's 77 districts across Nepal's three agroecological zones of mountains, hills and plains (i.e., terai) (Cunningham et al., 2017). *Suaahara* provided integrated programming across multiple sectors: nutrition; health and family planning services and WASH. Communities that were food‐insecure and had a higher prevalence of households disadvantaged socioeconomically or due to physical remoteness received additional interventions and support, including agriculture or enhanced homestead food production. *Suaahara* intended to improve household nutrition, health and hygiene behaviours; increase the use of quality nutrition and health services; increase the production and consumption of diverse and nutritious foods and strengthen nutrition governance at national and subnational levels. *Suaahara* used multiple social and behaviour change communication interventions—interpersonal communication, community events, mass media and m‐health—to improve household behaviours, complemented by capacity building to strengthen nutrition, health, WASH and agricultural services and governance, particularly at local levels (Appendices S1 and S2) (Anon, 2015; Cunningham & Kadiyala, 2013).

### 2.2 Design of the impact evaluation

The evaluation used a quasi‐experimental design with two arms (i.e., intervention and comparison) and two cross‐sectional surveys (i.e., baseline and endline) that were conducted in the same districts and clusters. The endline survey was originally planned for the end of *Suaahara* I but could not be conducted due to the 2015 Nepal earthquakes. The endline survey was postponed to 2020 and then delayed further due to the COVID‐19 pandemic. The endline survey was conducted in 2022, in the same season and sites, and by the same survey firm as was done for the 2012 baseline. Given changes since 2012 in programme design, interventions and context, the evaluation design was updated for the endline survey (Appendix S3).

The impact evaluation endline was conducted in four interventions and four comparison districts. Only eight districts could be used because half of the original comparison districts in the baseline survey became intervention districts as *Suaahara* scaled up. The intervention districts were Sindhupalchowk, Syangja, Rupandehi and Darchula whereas the pair‐matched comparison districts in corresponding order were Ramechhap, Tanahu, Chitwan and Jumla. Sampling at endline was done in 120 of the same old (i.e., pre‐federalism) wards as was done at baseline. At baseline, 17 households were sampled per old ward for a total sample of 2040. At endline, 20 households were sampled from each of 40 old wards, and 21 households were sampled from each of 80 old wards, for a total of 2480. The study is registered at clinicaltrials.gov with the identifier NCT05448287. Both the study protocol and statistical analysis plan were finalized on 11 October 2022 before the endline data were available for analysis.

The Research Ethics Committee of the Nepal Health Research Council in Nepal approved the baseline survey on 9 May 2012 and the endline survey on 21 July 2021. During data collection, written informed consent was obtained from the participants, assuring the confidentiality and anonymity of the information collected. All analyses were conducted with deidentified data, and security procedures to protect access to the data were taken.

### 2.3 Sample size and power

At baseline, 4080 households from 16 districts with children aged 0–5 years were surveyed. Because only half of the pairs of districts remained from baseline, the sample for the endline survey was adjusted to 2040 households. To be powered to answer the primary impact question separately for children under 2 years versus 2–5 years, the sample required was 2480 households, which equated to 310 households from each of the eight study districts: 255 with children 0–2 years and the remaining 55 with children between 2 and 5 years. Within these 310 households per district, all women of reproductive age (15–49 years) and all children 0–59 months were recruited for anthropometric measurements, and all women of reproductive age (15–49 years) and all children 6–59 months were recruited for measurement of haemoglobin and midupper arm circumference (MUAC). For weight and height, this sampling procedure aimed to yield at least 435 children of 0–5 years (i.e., 255 + 55 + 125) per district because 40% or more of the households sampled were expected to have another child aged 0–5 years.

Power calculations for the endline survey assumed four pairs (eight districts), 255 children aged 0–23.9 months and 55 children 24–59.9 months, per district, 90% power, two‐tailed testing, and *α* = 0.05. For weight and height, we assumed 435 children 0–5 years. To estimate the variance in models from the baseline survey, we included districts and village development committees as random effects and pairs as a fixed effect. Calculations of the minimal detectable effects accounted for the small number of degrees of freedom dictated by the number of districts. For three primary outcomes—child dietary diversity score (6–23.9 months), maternal dietary diversity score and child stunting (0–59.9 months)—the minimal detectable effects with 90% power were 0.330 food groups, 0.442 food groups and 15.9 percentage points, respectively (Appendix S4).

### 2.4 Sampling procedure

Within each ward, households were randomly selected for inclusion in the survey. In each ward, the supervisor and enumerators listed all households with details on child(ren) under 5 years of age. From this list, the supervisor circled the households with children under 2 years of age and 2–5 years of age, from which sample households were randomly selected. Diet and anthropometry (except MUAC and haemoglobin for children ≥6 months of age) of mothers and all children 6–59.9 months were measured. Twins were not included except for anthropometry and MUAC and haemoglobin (children ≥6 months of age).

### 2.5 Data collection

Three questionnaires were administered for three different respondents from late June to early September 2022: (1) mother of the child under 5 years of age, (2) household decision‐maker (male, when available) and (3) grandmother of the child under 5 years of age (when residing in the same household). This article reports on responses from mothers. All the questionnaires at endline were based on the ones designed in 2012 to enable comparison over time, but an extensive exposure module was developed to capture awareness of and participation in *Suaahara* activities. These questionnaires were vetted with the programme team to ensure interventions and steps (e.g., intermediate outcomes) along the programme impact paths were captured. The three respondents answered similar, but slightly different questionnaires (Appendix S5). The questionnaires included a module on exposure to *Suaahara* and other sources of multi‐sectoral nutrition‐related information and interventions. Outcomes were measured on individuals. Information on the altitude, latitude, and longitude (GPS coordinates) of all sampled households was collected using a Garmin eTrex 30x machine.

A local survey firm, New Era, conducted the baseline and endline surveys. New Era hired the field staff including quality controllers, field supervisors, anthropometrists and enumerators based on their prior work experience, academic qualifications (at least bachelors' degree), fluency in local languages and rapport‐building skills while keeping gender and caste/ethnicity diversity in mind. More enumerators than needed were invited to the training, with the best enumerators selected as data collectors. Training included sessions on survey objectives, listing of households, sample selection, obtaining consent, maintaining confidentiality and ethical standards, and questionnaire details. Training included practice sessions for interviewing, data recording on tablets, uploading data to the server, GPS use, quality control and roles and responsibilities of field team members.

Anthropometrists were trained through in‐depth lectures, discussions, and practice sessions on measurement of height/length, weight, MUAC and haemoglobin using anthropometry equipment, scales and HemoCue; recording of measurements; safety procedures; results of anthropometry and anaemia testing and sharing results with survey respondents. The procedure manual provided detailed steps on anthropometric measurement and haemoglobin testing. Anthropometry standardization was conducted separately for height/length and MUAC for children 0–23 months, children 24–59 months and women of reproductive age (15–49 years).

### 2.6 Outcomes

The primary study outcomes were (1) child dietary diversity score (mean), (2) maternal dietary diversity score (mean), (3) stunting prevalence among children under 5 years of age, (4) underweight prevalence among children under 5 years of age, (5) wasting prevalence among children under 5 years of age, (6) maternal underweight prevalence, (7) maternal anaemia prevalence and (8) anaemia prevalence among children aged 6–59.9 months. Among the secondary outcomes were (9) child minimum dietary diversity (prevalence), (10) maternal minimum dietary diversity (prevalence), (11) height‐for‐age (mean), (12) weight‐for‐age (mean), (13) weight‐for‐height (mean), (14) maternal body mass index (BMI) (mean), (15) maternal haemoglobin (mean), (16) child haemoglobin (mean), (17) infant and young child feeding practices among mothers and (18) health and WASH practices among mothers.

Dietary diversity scores for children between 6 and 59.9 months of age were calculated from 24‐h dietary recall data collected in the mothers' survey on seven food groups: (1) grains, white roots and tubers, and plantains; (2) legumes and nuts; (3) dairy; (4) meat, poultry and fish; (5) eggs; (6) vitamin‐A rich fruits and vegetables and (7) other fruits and vegetables. Minimum dietary diversity for children aged 6–59.9 months was defined as children eating foods from at least four of these seven food groups (World Health Organization Department of Child and Adolescent Health and Development, 2008). Minimum meal frequency was defined as consuming solid, semisolid or soft foods at least the minimum number of times in the previous day and was assessed only for children aged 6–23.9 months. A minimum acceptable diet, that is, consuming minimum dietary diversity and minimum meal frequency on the previous day, was assessed only for children aged 6–23.9 months. Dietary diversity scores for mothers were calculated using the Individual Dietary Diversity Score instrument that categorizes diets into 10 categories: (1) starchy staples; (2) beans, lentils and nuts; (3) dairy; (4) meat and poultry; (5) eggs; (6) fish; (7) dark green leafy vegetables; (8) vitamin‐A rich fruits and vegetables; (9) other vegetables and (10) other fruits. Minimum dietary diversity for mothers was defined as the intake of foods from at least 5 of the 10 food groups during the previous 24 h (Food and Agriculture Organization, 2021).

Anthropometric measures included the weight and height of mothers and weight and height or length of children. Child height (or length if under 2 years) was measured using a Shorr board. Haemoglobin levels were assessed for mothers and children. Each anthropometric measurement was taken twice, and the average of the measurements was calculated for each measure. Child stunting was calculated using height‐for‐age *z*‐scores (HAZ), with stunting defined as HAZ < −2. Child underweight was calculated using weight‐for‐age *z*‐scores (WAZ), with underweight defined as WAZ < −2. Child wasting was calculated using weight‐for‐height *z*‐scores (WHZ), with wasting defined as WHZ < −2. Child (6–59.9 months) anaemia was calculated based on children's haemoglobin levels adjusted for altitude and was defined as haemoglobin levels <11 g/dL. Maternal underweight was defined by BMI <18.5 kg/m^2^. Maternal anaemia was calculated based on mothers' haemoglobin levels adjusted for altitude and pregnancy. For pregnant women, anaemia was defined as haemoglobin levels <11 g/dL and for nonpregnant women, anaemia was defined as haemoglobin levels <12 g/dL.

Exclusive breastfeeding was defined as feeding the infant only breast milk until the child is 6 months of age and was measured only among children in the sample who were younger than 6 months of age. Early initiation of breastfeeding was defined as the initiation of breastfeeding within 1 h of birth and was measured only among children in the sample who were younger than 24 months of age. Introduction to complementary feeding was defined by the introduction of all types of complementary foods during the appropriate age window of 6–8.9 months. Sick child feeding was defined as offering more food (including breastmilk) than usual during the child's illness and was only measured among children 6–23.9 months who had an illness in the 2 weeks before data collection. An indicator of whether oral rehydration solution or zinc was administered in the presence of diarrhoea was used for children 6–23.9 months.

Adequate antenatal care was defined as having at least four or more antenatal care visits during pregnancy. Adequate post‐natal care was defined as having at least three post‐natal care visits in the week after delivery. Appropriate iron and folic acid supplementation was defined as taking supplementation tablets for at least 180 days during pregnancy. The use of modern family planning methods was defined as using female sterilization, male sterilization, intrauterine contraceptive device, implants, injectable, pills or condoms. Institutional delivery was defined as the delivery of the child at a health facility. Having had a handwashing station with soap and water was measured by observation. Handwashing at six critical times was measured as mothers' usually practicing handwashing at six critical times. Appropriate treatment methods for drinking water were boiling, adding bleach or chlorine, using a water filter (ceramic, sand and composite) or using solar disinfection (Sodis method) and was indicated by a household always engaging in these appropriate methods, without using any inappropriate treatment methods.

### 2.7 Statistical analysis

Analyses were done using Stata version 17 (StataCorp.). Data were checked for the assumption of normal distribution when relevant. Linear and logistic regression models were used to assess impact with the dependent variable for each model being an outcome. The models compared changes in each outcome from baseline to endline between the intervention and comparison arms of the study (Gertler et al., 2016). The impact was estimated and tested using intent‐to‐treat regression models in which survey (baseline vs. endline), arm (comparison vs. intervention) and their interaction were fixed effects. The interaction term estimated and tested impact as the difference in differences. The analyses accounted for clustering by district, ignoring the old wards, using a cluster sandwich estimator of the standard errors with data at the household level. This analytic method was appropriate because districts were assigned to receive either the intervention or be in the comparison arm, and the variation among districts within arms and the number of districts determined the standard errors and *p* values used for testing difference in differences. Estimates and *p* values are presented (Amrhein et al., 2019; Wasserstein et al., 2019) without adjustment for multiple comparisons, reflecting guidance for analyzing complex interventions (Leroy et al., 2022). Analyses were done for children 0–59.9 months combined and then stratified by three age categories: 0–5.9, 6‐23.9 and 24‐59.9 months. Sample sizes for these three age categories were at baseline 201, 702 and 1137 and at endline 453, 1587 and 440 at endline, respectively. The differences in sample sizes between baseline and endline resulted from the greater priority given to the 6‐23.9 age category at endline.

We also conducted exploratory stratified analyses to examine whether impact differed by socioeconomic status. Socioeconomic status was measured by an index constructed with principal components analysis using household asset items that were collected in both the baseline and endline surveys: lighting (improved), cooking (improved) and ownership of radio, TV, land, computer, refrigerator, table and chair, sofa, cupboard, clock, invertor, mobile phone, rickshaw, motorized vehicle and cart. The index was calculated separately at baseline and endline and divided into quintiles. Low socioeconomic status was defined as the two lower quintiles and high as the higher three quintiles for stratified analyses.

## 3 RESULTS

At baseline, the age of mothers and children and the distribution of caste/ethnicity in the sample were similar for comparison and intervention districts (Table 1). Comparison districts had lower socioeconomic status at baseline than intervention districts with 52.0% and 31.5% in the lowest two quintiles, respectively. Similar differences were seen at endline. The comparison arm had two districts in hills and one each in terai and mountains whereas the intervention arm had two districts in mountains and one each in terai and Hills.

**Table 1:** Sample demographics of comparison and intervention districts at baseline and endline.

Demographic variables	Comparison districts		Intervention districts	
	Baseline (*N* = 1020), mean (SD) or %	Endline (*N* = 1240), mean (SD) or %	Baseline (*N* = 1020), mean (SD) or %	Endline (*N* = 1240), mean (SD) or %
Mothers' age (years)	26.67 (6.39)	25.84 (5.37)	26.85 (5.98)	26.22 (5.46)
Child age (months)	27.14 (16.82)	16.63 (12.65)	28.30 (16.69)	16.96 (12.70)
0–5.9	10.78%	19.60%	8.92%	16.94%
6–23.9	34.90%	62.66%	33.92%	65.32%
24–59	54.31%	17.74%	57.16%	17.74%
Child sex: Female	46.27%	44.68%	46.76%	46.69%
Caste/ethnicity				
Socially excluded	42.55%	46.05%	43.04%	42.34%
Brahmin/Chhetri	46.18%	42.98%	45.29%	39.68%
Other	11.27%	10.97%	11.67%	17.98%
Wealth quintile				
Quintile 1 (lowest)	27.06%	25.32%	13.84%	14.76%
Quintile 2	24.90%	21.29%	17.66%	18.63%
Quintile 3	16.18%	21.29%	20.90%	20.81%
Quintile 4	15.69%	16.53%	24.53%	21.37%
Quintile 5 (highest)	16.18%	15.56%	23.06%	24.44%
Agroecological zone				
Terai	25%	25%	25%	25%
Hills	50%	50%	25%	25%
Mountains	25%	25%	50%	50%

### 3.1 Anthropometry

Stunting was reduced in the overall sample between baseline and endline by about 21 percentage points for both intervention and comparison districts, with no difference in reduction between the intervention and comparison districts (Table 2). For the overall sample, there was a reduction in HAZ of 0.94 in intervention districts and 0.82 in comparison districts. For children aged 0–5.9 months, HAZ improved 0.761 *z*‐scores more in intervention districts than in comparison districts (*p* = 0.004) (Table 3).

**Table 2:** Diet, nutritional status, infant and young child feeding, maternal health and WASH practices for the overall sample.

Outcomes	Comparison districts		Intervention districts		Difference in differences	
	Baseline, mean (SD) or %	Endline, mean (SD) or %	Baseline, mean (SD) or %	Endline, mean (SD) or %	Coefficient (SE) or percentage points	*p* Value
Stunting (H/LAZ < −2)	43.51	22.40	40.47	19.60	0.24	0.757
Underweight (WAZ < −2)	33.66	14.41	33.14	14.19	0.30	0.978
Wasting (W/LAZ < −2)	8.91	6.38	13.75	8.64	−2.58	0.629
HAZ	−1.75 (1.31)	−0.93 (1.39)	−1.75 (1.12)	−0.81 (1.41)	0.125 (0.132)	0.375
WAZ	−1.53 (1.06)	−0.73 (1.23)	−1.64 (1.05)	−0.80 (1.19)	0.039 (0.133)	0.780
WHZ	−0.73 (1.00)	0.32 (1.21)	−0.90 (1.04)	−0.56 (1.14)	−0.071 (0.155)	0.662
Maternal underweight (BMI < 18.5)	19.82	12.98	25.59	10.32	−8.43	<0.001
Maternal BMI	20.68 (2.70)	22.40 (6.70)	20.45 (2.88)	22.73 (3.77)	0.571 (0.546)	0.330
Child dietary diversity score (7 food groups, 6–59.9 months)	3.51 (1.13)	3.81 (0.99)	3.62 (1.12)	4.00 (1.06)	0.070 (0.14)	0.619
Child minimum dietary diversity (≥4 of 7 groups for 6–59.9 months)	49.01	62.59	56.51	70.00	−0.09%	0.882
Maternal dietary diversity score (9 food groups)	3.80 (1.21)	3.82 (1.05)	3.94 (1.05)	4.17 (1.09)	0.211 (0.22)	0.372
Maternal minimum dietary diversity (≥5 of 9 groups)	26.76	23.47	28.04	36.77	12.02	0.167
Child anaemia (6–59.9 months)	55.32	59.28	48.87	61.07	8.24	0.143
Child haemoglobin	10.50 (1.98)	11.67 (10.09)	10.96 (1.41)	11.44 (9.19)	−0.694 (0.458)	0.174
Maternal anaemia	38.54	36.61	37.78	34.52	−1.33	0.816
Maternal haemoglobin	12.25 (1.59)	12.45 (3.71)	12.33 (1.57)	12.81 (6.17)	0.268 (0.354)	0.474
Exclusive breastfeeding (0–5.9 months only)	57.27	57.61	50.00	60.00	9.66	0.422
Early initiation of breastfeeding (0–24 months only)	46.57	67.00	35.01	67.33	11.89	0.307
Introduction of complementary feeding (6–23.9 months only)	18.70	28.48	12.12	32.20	10.30	0.154
Sick child feeding (among children who had illness)	25.07	31.38	15.56	32.44	10.57	0.025
Minimum meal frequency (6‐23.9 months only)	78.93	80.95	66.76	83.83	15.05	0.004
Minimum acceptable diet (6‐23.9 mo only)	39.33	48.13	32.28	55.43	14.35	0.022
Adequate antenatal care (0–23.9 months only)	62.88	85.82	69.34	91.94	−0.34	0.485
Adequate post‐natal care (0–23.9 months only)	14.38	24.31	17.16	34.71	7.62	0.300
IFA supplementation (0–23.9 months only)	38.63	56.15	35.93	58.45	5.00	0.557
Modern family planning	33.57	35.40	38.96	35.81	−4.98	0.405
Institutional delivery (0–23.9 months only)	48.93	81.18	42.76	90.49	15.48	0.074
Handwashing station with soap and water	40.51	80.08	34.77	88.29	13.95	0.067
Handwashing at 6 critical times (mothers)	Not assessed	23.31	Not assessed	23.06	−0.25	0.980
Appropriate drinking water treatment methods (observation)	5.69	21.13	5.78	28.23	7.01	0.371

*Note*: Differences in differences were estimated using intent‐to‐treat regression models in which survey year, arm and their interaction were fixed effects, accounting for district clustering using a sandwich estimator of standard errors, with the interaction estimating differences between arms in changes over time.

Abbreviations: BMI, body mass index, H, height; HAZ, height‐for‐age *z*‐scores; IFA, iron–folic acid; LAZ, length for age *z*‐score; W, weight; WASH, water, sanitation and hygiene; WAZ, weight‐for‐age *z*‐scores; WHZ, weight‐for‐height *z*‐score.

**Table 3:** Diet, nutritional status, infant and young child feeding, maternal health and WASH practices for respondents with children between 0 and 5.9 months of age.

Outcomes	Comparison districts		Intervention districts		Difference in differences	
	Baseline, mean (SD) or %	Endline, mean (SD) or %	Baseline, mean (SD) or %	Endline, mean (SD) or %	Coefficient (SE) or percentage points	*p* Value
Stunting (H/LAZ < −2)	10.48	6.36	10.99	5.77	−1.10	0.786
Underweight (WAZ < −2)	13.89	5.39	16.48	5.24	−2.74	0.735
Wasting (W/LAZ < −2)	6.54	2.99	6.98	6.37	2.94	0.447
HAZ	−0.45 (1.28)	−0.11 (1.29)	−0.77 (1.14)	0.33 (1.51)	0.761 (0.18)	0.004
WAZ	0.70 (1.12)	−0.06 (1.11)	−1.00 (1.15)	−0.01 (1.20)	0.355 (0.255)	0.206
WHZ	0.38 (1.11)	0.03 (1.18)	−0.46 (1.20)	−0.37 (1.25)	−0.313 (0.310)	0.347
Maternal underweight (BMI < 18.5)	8.18	8.23	23.08	4.29	−18.84	<0.001
Maternal BMI	21.24 (2.27)	23.23 (12.85)	20.87 (2.92)	23.25 (3.43)	0.391 (1.169)	0.748
Maternal dietary diversity score (9 food groups)	3.78 (1.28)	3.88 (1.18)	4.0 (1.15)	4.29 (1.20)	0.187 (0.24)	0.470
Maternal minimum dietary diversity (≥5 of 9 groups)	30.91	25.51	32.97	40.48	12.9	0.209
Maternal anaemia	50.91	34.16	36.26	33.33	13.82	0.136
Maternal haemoglobin	11.98 (1.59)	12.75 (5.65)	12.49 (1.58)	12.34 (1.44)	‐0.913 (0.549)	0.140
Exclusive breastfeeding	57.27	57.61	50.00	60.00	9.66	0.422
Early initiation of breastfeeding	49.09	68.05	29.67	64.90	16.27	0.190
Sick child feeding (among children who had illness)	28.95	24.39	16.13	26.67	15.10	0.281
Adequate antenatal care	59.09	85.96	72.53	93.20	‐6.20	0.718
Adequate post‐natal care	13.64	25.51	20.88	39.05	6.30	0.834
IFA supplementation	36.36	52.67	32.97	58.85	9.57	0.337
Modern family planning	1.82	11.93	14.29	17.14	‐7.26	0.051
Institutional delivery	53.64	85.19	57.47	90.95	1.93	0.487
Handwashing station with soap and water	37.61	79.25	31.11	87.62	14.87	0.034
Handwashing at 6 critical times (mothers)	Not assessed	20.58	Not assessed	19.52	‐1.06	0.910
Appropriate drinking water treatment methods (observation)	4.55	22.22	5.49	25.71	3.49	0.990

*Note*: Differences in differences were estimated using intent‐to‐treat regression models in which survey year, arm and their interaction were fixed effects, accounting for district clustering using a sandwich estimator of standard errors, with the interaction estimating differences between arms in changes over time.

Abbreviations: BMI, body mass index, H, height; HAZ, height‐for‐age *z*‐scores; IFA, iron–folic acid; LAZ, length for age *z*‐score; W, weight; WASH, water, sanitation and hygiene; WAZ, weight‐for‐age *z*‐scores; WHZ, weight‐for‐height *z*‐score.

Underweight was reduced in the overall sample between baseline and endline by about 19 percentage points for both intervention and comparison districts, with no difference in reduction between intervention and comparison districts (Table 2). For the overall sample, intervention districts had a reduction of about 0.84 *z*‐scores in WAZ, and comparison districts had a reduction of about 0.80 *z*‐scores.

Wasting was reduced in the overall sample between baseline and endline for both intervention and comparison districts, with little difference in this reduction between intervention and comparison districts. For the overall sample, intervention districts had a reduction of about 0.34 *z*‐scores in WAZ, and comparison districts had a reduction of about 0.41 *z*‐scores.

The difference in differences for maternal underweight in the overall sample was −8.45 percentage points (*p* < 0.001), that is, intervention districts reduced maternal underweight by 8.45 percentage points more than comparison districts. This effect was consistent across mothers of the three child age categories (Tables 3, 4, 5), with the largest difference in differences being −18.84 percentage points (*p* < 0.001) among mothers of children aged 0–5.9 months. There were minor differences in maternal BMI between baseline and endline in both intervention and comparison districts (Table 2). For the overall sample, intervention districts had an average increased BMI of about 2.28 units, and comparison districts had an average increased BMI of about 1.72 units (*p* = 0.330).

**Table 4:** Diet, nutritional status, infant and young child feeding, maternal health and WASH practices for respondents with children between 6 and 23.9 months of age.

Outcomes	Comparison districts		Intervention districts		Difference in differences	
	Baseline, mean (SD) or %	Endline, mean (SD) or %	Baseline, mean (SD) or %	Endline, mean (SD) or %	Coefficient (SE) or percentage points	*p* Value
Stunting (H/LAZ < −2)	39.83	25.19	37.39	21.66	−1.09	0.584
Underweight (WAZ < −2)	33.99	16.54	31.88	15.31	0.88	0.981
Wasting (W/LAZ < −2)	11.83	7.15	17.89	9.08	−4.13	0.565
HAZ	−1.67 (1.24)	−1.05 (1.38)	−1.68 (1.13)	−0.97 (1.30)	0.090 (0.148)	0.562
WAZ	−1.57 (1.00)	−0.81 (1.23)	−1.57 (1.16)	−0.90 (1.16)	−0.090 (0.146)	0.557
WHZ	−0.95 (0.95)	−0.37 (1.20)	−0.93 (1.18)	−0.58 (1.13)	−0.223 (0.173)	0.238
Maternal underweight (BMI < 18.5)	20.85	14.54	28.32	11.85	−10.16	0.008
Maternal BMI	20.70 (2.93)	22.09 (3.96)	20.24 (2.78)	22.53 (3.70)	0.903 (0.531)	0.133
Child dietary diversity score (7 food groups)	3.40 (1.18)	3.78 (0.99)	3.27 (1.23)	3.95 (1.07)	0.294 (0.140)	0.072
Child minimum dietary diversity (≥4 of 7 groups)	46.07	61.90	43.06	68.40	9.51	0.028
Maternal dietary diversity score (9 food groups)	3.87 (1.19)	3.84 (1.01)	4.00 (1.04)	4.15 (1.07)	0.185 (0.210)	0.396
Maternal minimum dietary diversity (≥5 of 9 groups)	28.09	23.29	30.06	35.93	10.67	0.258
Child anaemia (6–23.9 months)	75.93	65.38	65.90	68.40	13.05	0.071
Child haemoglobin	9.85 (1.90)	11.45 (10.06)	10.44 (1.32)	11.36 (9.87)	−0.679 (0.512)	0.226
Maternal anaemia	39.55	38.35	41.91	34.20	−6.51	0.292
Maternal haemoglobin	12.13 (1.66)	12.39 (3.38)	12.22 (1.42)	13.04 (7.54)	0.565 (0.394)	0.195
Early initiation of breastfeeding	45.79	66.67	36.42	67.96	10.66	0.366
Introduction of complementary feeding	18.70	28.48	12.12	32.20	10.30	0.154
Sick child feeding (among children who had illness)	28.95	34.39	12.59	34.77	16.74	0.002
Oral rehydration solution/zinc for diarrhoea	20.6	8.0	7.0	11.8	17.4	0.057
Minimum meal frequency	78.93	80.95	66.76	83.83	15.05	0.004
Minimum acceptable diet	39.33	48.13	32.28	55.43	14.35	0.022
Adequate antenatal care	64.04	85.77	68.50	91.61	1.38	0.429
Adequate post‐natal care	14.61	23.94	16.18	33.58	8.07	0.210
IFA supplementation	39.33	57.24	36.71	58.34	3.72	0.655
Modern family planning	27.91	40.28	32.93	39.88	−5.42	0.294
Institutional delivery	47.47	79.92	39.00	90.37	18.92	0.048
Handwashing station with soap and water	43.94	80.00	36.71	88.86	16.09	0.081
Handwashing at 6 critical times (mothers)	Not assessed	24.58	Not assessed	23.09	−1.49	0.870
Appropriate drinking water treatment methods (observation)	5.34	20.85	8.09	30.00	6.40	0.911

*Note*: Differences in differences were estimated using intent‐to‐treat regression models in which survey year, arm and their interaction were fixed effects, accounting for district clustering using a sandwich estimator of standard errors, with the interaction estimating differences between arms in changes over time.

Abbreviations: BMI, body mass index, H, height; HAZ, height‐for‐age *z*‐scores; IFA, iron–folic acid; LAZ, *length for age z‐score*; W, weight; WASH, water, sanitation and hygiene; WAZ, weight‐for‐age *z*‐scores; WHZ, weight‐for‐height *z*‐score.

**Table 5:** Diet, nutritional status, infant and young child feeding, maternal health and WASH practices for respondents with children between 24 and 59.9 months of age.

Outcomes	Comparison districts		Intervention districts		Difference in differences	
	Baseline, mean (SD) or %	Endline, mean (SD) or %	Baseline, mean (SD) or %	Endline, mean (SD) or %	Coefficient (SE) or percentage points	*p* Value
Stunting (H/LAZ < −2)	52.30	29.95	46.97	25.11	0.49	0.898
Underweight (WAZ < −2)	37.32	16.82	36.49	18.64	2.65	0.482
Wasting (W/LAZ < −2)	7.48	7.31	12.31	9.13	−3.01	0.422
HAZ	−2.06 (1.19)	−1.41 (1.12)	−1.96 (1.02)	−1.29 (1.16)	0.011 (0.161)	0.950
WAZ	−1.66 (1.00)	−1.18 (1.04)	1.78 (0.91)	−1.18 (0.98)	0.110 (0.083)	0.230
WHZ	−0.66 (0.99)	−0.53 (1.21)	−0.94 (0.91)	−0.68 (1.06)	0.136 (0.125)	0.310
Maternal underweight (BMI < 18.5)	21.48	12.73	24.36	10.45	−5.16	0.083
Maternal BMI	20.56 (2.61)	22.55 (3.85)	20.50 (2.93)	22.95 (4.24)	0.461 (0.604)	0.471
Child dietary diversity score (7 food groups)	3.58 (1.09)	3.91 (1.01)	3.83 (0.99)	4.17 (0.99)	0.007 (0.15)	0.965
Child minimum dietary diversity (≥4 of 7 groups)	50.90	65.00	64.49	75.91	−2.68	0.914
Maternal dietary diversity score (9 food groups)	3.77 (1.20)	3.70 (1.01)	3.90 (1.04)	4.15 (1.05)	0.310 (0.20)	0.158
Maternal minimum dietary diversity (≥5 of 9 groups)	25.09	21.82	26.07	36.36	13.56	0.090
Child anaemia (24–59.9 months)	42.10	37.73	38.77	34.19	−0.21	0.916
Child haemoglobin	10.92 (1.93)	12.46 (10.17)	11.27 (1.37)	11.72 (6.02)	−1.09 (0.751)	0.191
Maternal anaemia	35.44	33.18	35.57	36.82	3.51	0.657
Maternal haemoglobin	12.38 (1.53)	12.34 (1.35)	12.38 (1.65)	12.37 (1.57)	0.026 (0.269)	0.925
Sick child feeding (among children who had illness)	21.77	26.83	17.92	26.15	3.17	0.698
Modern family planning	44.10	44.09	46.77	38.64	−8.12	0.391
Handwashing station with soap and water	38.88	81.28	34.19	86.82	10.23	0.049
Handwashing at 6 critical times (mothers)	Not assessed	21.82	Not assessed	26.36	4.54	0.699
Appropriate drinking water treatment methods (observation)	6.14	20.91	4.46	24.09	4.86	0.351

Differences in differences were estimated using intent‐to‐treat regression models in which survey year, arm, and their interaction were fixed effects, accounting for district clustering using a sandwich estimator of standard errors, with the interaction estimating differences between arms in changes over time.

Abbreviations: BMI, body mass index, H, height; HAZ, height‐for‐age *z*‐scores; LAZ, *length for age z‐score*; W, weight; WASH, water, sanitation and hygiene; WAZ, weight‐for‐age *z*‐scores; WHZ, weight‐for‐height *z*‐score.

### 3.2 Dietary diversity

Child dietary diversity score and minimum dietary diversity in the 6–23.9 months age group improved more in intervention districts than comparison districts by 0.294 food groups (*p* = 0.072) and 9.51 percentage points (*p* = 0.028), respectively (Table 4). Maternal minimum dietary diversity in the 24–59.9 age group improved more in intervention districts than in comparison districts by 13.56 percentage points (*p* = 0.090) (Table 5).

### 3.3 Anaemia

Child anaemia increased by 13.05 percentage points (*p* = 0.071) in the intervention districts relative to the comparison districts for children 6–23.9 months of age (Table 4). There were minor differences in child haemoglobin between baseline and endline in both intervention and comparison districts. For the overall sample, there was no substantial difference between intervention and comparison districts; intervention districts had an average increased haemoglobin of about 0.48 g/dL, and comparison districts had an average increased haemoglobin level of about 1.17 g/dL (*p* = 0.174) (Table 2).

There were no differences in maternal anaemia prevalence between intervention and comparison districts from baseline to endline. For the overall sample, there was no substantial difference between intervention and comparison districts; intervention districts had an average increased haemoglobin of about 0.48 g/dL, and comparison districts had an average increased haemoglobin level of about 0.20 g/dL (*p* = 0.474) (Table 2).

### 3.4 Infant and young child feeding

A moderate increase (10 percentage points) in the prevalence of children being exclusively breastfed was observed in intervention districts from baseline to endline, while this prevalence remained almost identical at baseline and endline in comparison districts (Table 2). The *p* value for the difference in differences between intervention and comparison districts was 0.422. For early initiation of breastfeeding in the overall sample, there was an increase of about 20 percentage points for comparison districts and 32 percentage points for intervention districts between baseline and endline (*p* = 0.307 for the difference in differences). For the introduction to complementary feeding in the overall sample, there was an increase of about 10 percentage points for comparison districts and 20 percentage points for intervention districts (*p* = 0.154 for the difference in differences).

Sick child feeding for the overall sample increased by 10.57 percentage points (*p* = 0.025) in intervention districts relative to comparison districts. This effect was largest for children aged 6–23.9 months with a difference in differences of 16.74 percentage points (*p* = 0.002) (Table 4). For administering oral rehydration solution or zinc to children 6–23.9 months, intervention districts increased by 17.4 percentage points (*p* = 0.057) relative to comparison districts. Within this sample, the difference in differences for having an appropriate minimum meal frequency and minimum acceptable diet was 15.05 percentage points (*p* = 0.004) and 14.35 percentage points (*p* = 0.022), respectively.

### 3.5 Maternal health and WASH practices

For adequate antenatal care (≥4 visits) in the overall sample of mothers with children 0–23.9 months, both intervention and comparison districts increased from baseline to endline about 23 percentage points (Table 2). Adequate post‐natal care (≥3 visits) in mothers with children 0–23.9 months increased about 10 percentage points in comparison districts and 18 percentage points in intervention districts (*p* = 0.300 for the difference in differences). Adequate iron and folic acid supplementation during pregnancy (≥180 days) in the overall sample increased about 18 percentage points in comparison districts and 23 percentage points in intervention districts (*p* = 0.557 for the difference in differences).

For modern methods of family planning among mothers of children aged 0–5.9 months, the difference in differences was −7.26 percentage points (*p* = 0.051), a reduction in the use of modern family planning in intervention districts relative to comparison districts. The increase in institutional delivery was larger in intervention districts, with a difference in differences for the overall sample of 15.5 percentage points (*p* = 0.074); among mothers of children aged 6–23.9 months the difference in differences was 18.92 percentage points (*p* = 0.048).

The availability of handwashing stations with soap and water doubled or more in both comparison and intervention districts. The difference was larger in intervention districts, and the difference in differences for the overall sample was 13.95 percentage points (*p* = 0.067). The difference in differences was 14.87 percentage points (*p* = 0.034) for mothers of children aged 0–5.9 months, 16.09 percentage points (*p* = 0.081) for mothers of children aged 6–23.9 months and 10.23 percentage points (*p* = 0.049) for mothers of children aged 24–59.9 months. The baseline survey did not have data on handwashing at all critical times; at endline, there was no difference between comparison and intervention districts, with about 1 in 4 mothers practicing handwashing at 6 critical times. Appropriate drinking water treatment methods at all times increased from baseline to endline in both the comparison and intervention districts (*p* = 0.371 for the difference in differences).

### 3.6 Socioeconomic equity gap

The gaps in maternal minimum dietary diversity and child minimum acceptable diet between those households with low (i.e., two lowest quintiles) and high (i.e., three highest quintiles) socioeconomic status were reduced in the intervention districts by 11.4 (*p* = 0.086) and 9.7 percentage points (*p* = 0.002), respectively, whereas no reductions in gaps were seen in the comparison districts (Appendix S6). The socioeconomic gap in early initiation of breastfeeding was reduced in the intervention districts by 16.8 percentage points (*p* = 0.008) and similarly in the comparison districts by 19.8 percentage points (*p* = 0.084).

## 4 DISCUSSION

Substantial impacts on multiple maternal and child outcomes were plausibly attributable to the *Suaahara* interventions. The impacts were seen primarily and most consistently in maternal nutrition and in complementary feeding practices of children 6–23.9 months of age who had the greatest potential to benefit.

The intervention districts, relative to comparison districts, reduced maternal underweight by 8.45 percentage points overall, with a greater reduction in mothers of the youngest children. These reductions in maternal underweight were consistent with improvements in maternal and fetal condition that were intended by the Suaahara interventions and that were manifested in the early post‐natal period as a greater linear size of 0.761 *z*‐scores of the infant 0 to 5.9 months.

Complementary feeding practices with children between 6 and 23.9 months of age improved more in intervention districts than comparison districts, including child dietary diversity by 0.294 food groups and minimum dietary diversity by 9.51 percentage points, feeding sick child more and administering oral rehydration solution and zinc for diarrhoea by about 17 percentage points each, and minimum meal frequency and minimum acceptable diet by 14–15 percentage points. These improvements were consistent with the content, focus and targeting of the *Suaahara* interventions on enhancing child nutrition for this age category through improving infant and young child feeding and health practices. These improvements were also consistent with evidence from *Suaahara* that interaction with frontline workers and participation in community events had positive associations with maternal dietary diversity and exposure to mass media had positive associations with child dietary diversity for children 6–23.9 and 24–59.9 months in a dose–response manner within and across intervention modalities; exposure to all three interventions (i.e., interaction with frontline workers, community events, and mass media), compared with fewer exposures, had the strongest association with maternal and child dietary diversity (Suresh et al., 2019).

Several outcomes improved in both intervention and comparison districts. For example, stunting reduced in both arms from baseline to endline from about 40% to 20%, a large reduction of about 2 percentage points per year. Other data support this trend in the reduction of stunting in children. For example, the 2022 Nepal Demographic and Health Survey shows that stunting reduced from 36% to 25% from 2016 to 2022. Exclusive breastfeeding was reduced from 66% to 56% during the same period, a reduction not seen in this study. Children having minimum dietary diversity increased from 47% to 78% and the prevalence of anaemia in women of reproductive age went from 41% to 34% (MoHP, Nepal New ERA, & ICF International Inc., 2023). Similar secular trends have been observed in other countries. This secular progress in nutrition and health outcomes is consistent with findings from other programmes in similar contexts. For example, for Alive & Thrive in Bangladesh, exclusive breastfeeding (Menon, Nguyen, Saha, Khaled, Kennedy, et al., 2016) and complementary feeding (Menon, Nguyen, Saha, Khaled, Sanghvi, et al., 2016) improved more in intensive areas than comparison areas, but child stunting prevalence was reduced in both intervention and comparison areas, reflecting the secular trend in child linear growth. Child anaemia increased in intervention districts relative to comparison districts for children 6–23.9 months of age primarily because of a nearly 10 percentage point decrease in comparison districts for reasons not known.

The exploratory analyses showed that differences between intervention and comparison districts can depend on socioeconomic status. For two outcomes, maternal minimum dietary diversity and child minimum acceptable diet, *Suaahara* reduced gaps in equity. These exploratory results suggest opportunities for further research into conditions that enable improved outcomes and how programme effects differ among sub‐populations, research that is needed to understand how best to improve equity (Global Nutrition Report, 2022).

This evaluation study used a quasi‐experimental plausibility design (Habicht, 1999) and a difference‐in‐differences analysis (Gertler et al., 2016). Since randomization of districts could not be done, matching was used at baseline to have similar intervention and comparison districts. Although at baseline comparison districts had lower socioeconomic status than did intervention districts, most outcomes were similar between comparison and intervention districts at baseline. The difference‐in‐differences analyses accounted for baseline differences under the assumption that no other major interventions or events besides *Suaahara* occurred differently between comparison and intervention districts during the period between the surveys. Field visits to check this assumption revealed that, although many activities with similar themes occurred in some comparison areas, no other interventions were conducted at the scale or intensity of the *Suaahara* programme to affect the results of the endline impact evaluation (Appendix S3). Data were collected in the same (i.e., rainy) season at both baseline and endline, yielding accurate estimates of impact, but if data had been collected in a different season at both baseline and endline, estimates of means and prevalence may have differed. Data collectors were well‐trained and some measurements (e.g., anthropometry) were objective. Other measurements relied on reporting from mothers which could have had reporting errors stemming from recall or social desirability; based on previous work in Bangladesh (Menon, Nguyen, Saha, Khaled, Sanghvi, et al., 2016), social desirability in reporting is unlikely to have caused bias.

Improvements in intervention districts are relative to comparison districts were demonstrated with plausible evidence for some outcomes despite the substantial challenges that Nepal and *Suaahara* faced between baseline (2012) and endline (2022) and the limitations in statistical power that resulted from the small number of districts that then could be included in the evaluation study. Implementation was affected by the 2015 major earthquakes; natural disasters such as recurring droughts, floods and landslides; a complete political restructuring to decentralize power and the severe acute respiratory syndrome coronavirus‐2 pandemic. These events affected programme reach, erased inputs given to communities and increased internal migration, all likely diluting the impact. Furthermore, the number of comparison districts that was planned at baseline was cut substantially by scaling up, limiting both the power to detect impacts for some outcomes and the opportunity for geographic sub‐analyses.

The *Suaahara* programme was long‐term and at scale, targeting a specific population. As such, new pregnancies occurred continually and children transitioned out of the 1000‐day period which required ongoing implementation to an evolving target population. Furthermore, *Suaahara* involved multiple sectors and was designed and implemented to improve many behaviours simultaneously. This programme design requires front‐line workers to attend to improving many behaviours rather than focusing on a small set of behaviours; future research should investigate this trade‐off. Other issues for future research are the value of adding agricultural intervention into the main *Suaahara* programme, learning about who was reached and exposed to the programme and impact on them, understanding variation in impact by intensity of programming and type of intervention, addressing socioeconomic and geographic inequity and examining the paths to impact.

## AUTHOR CONTRIBUTIONS

Edward A. Frongillo, Deependra K. Thapa, Shalini Suresh and Ramesh P. Adhikari performed the research. Edward A. Frongillo, Ramesh P. Adhikari, Kenda Cunningham, Pooja Pandey Rana and Rolf Klemm designed the research study. Edward A. Frongillo, Shalini Suresh and Ramesh P. Adhikari analyzed the data. Edward A. Frongillo and Shalini Suresh wrote the manuscript. All authors reviewed the manuscript draft, provided comments, and approved the final draft.

## CONFLICT OF INTEREST STATEMENT

The authors declare no conflict of interest. Kenda Cunningham, Pooja Pandey Rana, Ramesh P. Adhikari, Subir Kole, Bhim Pun, and Rolf Klemm were members of the program implementation team that designed and implemented the interventions discussed in this article; Debendra P. Adhikari was affiliated with the sponsor. They reviewed the manuscript and provided interpretation of the results, but final decisions for content were made by the primary authors from the evaluation team (Edward A, Frongillo, Shalini Suresh, and Deependra K. Thapa).

## Supporting information

Supporting information.

## Data Availability

The data that support the findings of this study are available from the corresponding author upon reasonable request.
