# Supplementary material for: Impact of Suaahara, an integrated nutrition programme, on maternal and child nutrition at scale in Nepal
Source: Matern Child Nutr. 2024 Feb 11;22(1):e13630. doi: 10.1111/mcn.13630 (PMC12647975; doi:10.1111/mcn.13630)
Supplement: Supplementary file 1 — Supporting information. [file MCN-22-e13630-s001.docx]

Appendix 1. Description of *Suaahara* implementation.

*Suaahara* facilitated improvement of nutrition policies at national and district levels, coordination among stakeholders, and training and support of a diverse set of governmental and non-governmental frontline workers in health, agriculture, and WASH to improve service quality (Cunningham et al., 2017). The government’s female community health volunteers were the primary means of engaging the program target populations with activities for social and behavior change communication through home visits and mothers' group meetings. In addition, the program partnered with district‐level non-governmental organizations that hired program field supervisors to support the government frontline workers. Other frontline workers that contributed to *Suaahara* programming were individuals and groups working with health, nutrition, WASH, and agriculture: health assistants, assistant health workers, assistant nurse midwifes; traditional healers; agricultural extension workers; livestock extension workers; social mobilizers; village development and WASH committees; citizen awareness centers; village development nutrition and food security steering committees; ward citizen forums; field supervisors; mothers' groups; peer facilitators; and community hygiene and sanitation facilitators. At the sub‐district level, activities for social and behavioral change communication on maternal, infant, and young child nutrition; maternal and child health and family planning; and WASH was implemented throughout *Suaahara* districts for all 1,000‐day households using mass media, community mobilization, and interpersonal communication. As of the end of *Suaahara* I. about 2.4 million people across 1,900 village development committees were reached, about a tenth of Nepal’s total population. *Suaahara* trained 33,688 volunteers and 32,784 additional frontline workers across maternal, infant, and young child nutrition, family planning, and WASH.

*Suaahara* undertook other activities in disadvantages areas (Cunningham et al., 2017). Materials for the construction of toilets and hand washing stations and for homestead food production were distributed. The program facilitated nutrition governance activities, such as discussion sessions at community citizens awareness centers and linked households with local ward citizen forums. Field supervisors gave priority to disadvantaged households for regular home visits to provide counseling, distribute agricultural inputs and advice, and assess access to and use of toilets.

**Appendix A: *S***

Increased income by selling HFP surplus

Leverage external agriculture resources

Homestead garden and backyard poultry established

Improved knowledge and skills of 1000-day women

Capacity building of 1000 day women on homestead gardens and poultry rearing

Vegetable seeds and poultry distributed for 1000-day families

Basic EHFP package for 1000-day families

Increased food security

Increased women empowerment

Increased production of diverse and nutrient rich foods

Increased resilience to shocks

HFP groups formed and active (monthly meetings, saving/credit activities, registration with GoN)

Improved quality of health, FP, and nutrition services at facility and community levels

Increased service coverage of health, FP and nutrition services at facility and community levels

Equipment and supplies procured for provision of health, FP and nutrition services by health workers and FCHV

Improved availability of health, FP and nutrition services at facility and community level, including SAM/MAM screening and follow-up

Capacity building of HW/FCHV through trainings and follow-up/onsite coaching for health, FP, and nutrition services (e.g. CB-IMNCI, MIYCN/NACS)

Equipment and supplies provided to health facilities and FCHVs

Improved HW/FCHVs knowledge and skills, including counseling, for health, FP and nutrition service provision at facility and community levels

Support to health system management and recording and reporting

Improved knowledge and skills among private sector actors on producing WASH materials locally and social marketing of WASH products

Increased access and availability of affordable WASH products

Improved WASH behaviors in the community

WASH Marts established

Program approach for engagement of social entrepreneurs and private sector actors developed

approaches and materials designed

Capacity building of local WASH private sector actors through trainings and follow-up/onsite coaching for WASH including branding, and social marketing

Establish linkages between rural and urban private sector actors

SMS messages sent to 1000-day families (and IVR, PSA, TV program)

Bhanchhin Aama broadcast via radio, FB, and YouTube

Home visits with 1000-day families

Improved health, FP, nutrition and WASH practices among 1000-day families

Increased health, FP, nutrition and WASH service-seeking by 1000-day families

Increased exposure to and knowledge on ideal health, FP, nutrition and WASH behaviors among 1000-day families

Increased awareness of health, FP, nutrition and WASH platforms and services among 1000-day families

Material inputs designed, distributed

Community events and platforms strengthened for 1000-day families

Media and technology approaches designed and produced

Improved political commitment towards nutrition-promoting actions among government stakeholders

Strengthened GoN program plans, training resources, and IEC materials with updated nutrition content integrated

Multi-sector coordination mechanisms (e.g. NFSSC, WASHCC etc.) functional at all levels

Increased engagement with local governments and demand for prioritization of nutrition needs

National adoption of revised health, FP, nutrition and wash training packages and materials

Improved cross-sector coordination at all levels for multi-sectoral nutrition

Increased inclusion of multi-sectoral nutrition in plans and policies

Increased budget allocation and utilization for multi-sectoral nutrition

Technical support and advocacy to multi-sector nutrition government stakeholders at provincial, municipal and ward level

Technical and financial support by each SII thematic team to national government to integrate nutrition across sectors

Formation and institutionalization of multi-sector nutrition committees (e.g. NFSSC, WASHCC) at all levels

Awareness and capacity strengthening of government stakeholders on health, FP, nutrition and WASH

Awareness raising among 1000-day families and communities on multi-sectoral nutrition and governance systems

## Appendix 2. Program Impact Pathway for Suaahara: Core+ Areas

Staff and volunteers hired, trained and mobilized: Technical thematic officers, Field Supervisors, Community Nutrition Volunteers, Peer Faciliators, Community WASH Volunteers, Village Model Farmers, GESI Champions, etc.

**Inputs**

**Process**

**Improved nutritional status of women and children**

**Monitoring, Evaluation and Research for Learning;** **Gender Equality and Social Inclusion approach; Coordination and Collaboration with Government stakeholder at all levels**

**Outputs**

**Impact**

**Outcomes**

Appendix 3. Updates to the evaluation design for the endline survey given changes since 2012 in program design, interventions, and context.

The baseline survey was conducted in 16 districts (eight intervention and eight comparison). The assignment to intervention versus comparison was done purposefully. The 8 intervention districts of the original 16 paired districts were chosen in consultation with the program team as they were going to be the early intervention districts and therefore have maximum time for intervention exposure. Then a series of characteristics including agro-ecological zone, east to west development zone, no planned Suaahara interventions, and similar socio-economic and demographic factors was used to select a matching district for each of these 8 intervention districts. Among these eight intervention-comparison pairs, however, four comparison districts became intervention districts, and one planned intervention district was dropped from being a Suaahara intervention district early on and thus only eight districts (four intervention and four comparison) remained for inclusion as matched pairs in the endline survey of the impact evaluation.

In 2017, Nepal underwent a restructuring of administrative and geographic boundaries including a transition from 75 to 77 districts organized into 7 provinces and 753 municipalities. Within the districts, rural and urban municipalities were allocated to replace and, in most instances, amalgamate the former village development committees as the first sub-district unit, with wards now being the smallest formal administrative unit. Thus, a detailed sub-national mapping was necessary to link former village development committees and wards from baseline to the newly restructured municipalities and wards.

Other government and non-government activities, as well as a spillover of *Suaahara* interventions into comparison areas, between 2012 and 2020 could have affected the impact evaluation results. Therefore, an intervention mapping exercise in the evaluation districts was done as part of the study design and questionnaire development. An initial field visit to the 8 districts revealed that while there were many activities in similar thematic areas, no other interventions was conducted at the scale or intensity of the *Suaahara* program to affect the results of the endline impact evaluation.

Appendix 4. Power calculation for impact evaluation endline household survey.

| Indicator | Variance (district) | Variance (individual) | Intraclass correlation | Standard deviation (individual) | Individuals per district | Effect size (Units) | Effect size (SD) |
| --- | --- | --- | --- | --- | --- | --- | --- |
| **Dietary diversity score (6-23.9m)** | 0.00085 | 1.92678 | 0.00044 | 1.38808 | 190 | 0.330 | 0.238 |
| **Dietary diversity score (maternal)** | 0.016511 | 1.073162 | 0.01515 | 1.03594 | 310 | 0.442 | 0.427 |
| **Child stunting**  **(0-5y)** | 0.00205 | 0.22711 | 0.00894 | 0.47656 | 435 | 0.159 | 0.334 |
| Child underweight  (0-5y) | 0.004023 | 0.205911 | 0.01916 | 0.45377 | 435 | 0.209 | 0.461 |
| Child wasting  (0-5y) | 0.00132 | 0.09371 | 0.01388 | 0.30613 | 435 | 0.123 | 0.400 |
| Maternal underweight  (0-5y) | 0.00402 | 0.20591 | 0.01916 | 0.45377 | 310 | 0.150 | 0.378 |
| Maternal anemia (0-5y) | 0.00057 | 0.21294 | 0.00267 | 0.46145 | 310 | 0.112 | 0.242 |
| Child anemia  (6-59m) | 0.00340 | 0.22424 | 0.01495 | 0.47354 | 370 | 0.198 | 0.418 |

**Note:** These calculations assumed 4 pairs (8 districts), 255 children aged 0-23.9 months and 55 children 24-59.9 months, per district, 90% power, 2-tailed, and alpha 0.05. For weight and height, we assumed that sampling all children in the household would yield at least 435 children 0-5 years (i.e., 255 + 55 + 125) per district because 40% or more of the households sampled would have another child aged 0-5 years. To estimate variance, the following were included in the models: districts and VDC as random effects and pair as a fixed effect. Calculations of the effect sizes accounted for the small number of degrees of freedom dictated by the number of districts. The indicators in bold were the primary outcomes for study registration.

Appendix 5. Questionnaire modules.

The three questionnaires differed in two ways. First, the household head (male when possible) questionnaire included questions on household demographics and socioeconomics, maternal and child health and nutrition, feeding practices, and WASH knowledge, household food security, land use and agricultural practices, various dimensions of empowerment, as well as observations on household water and sanitation practices. Second, the mother questionnaire included questions on maternal and child health and nutrition including antenatal care (ANC), postnatal care (PNC), newborn care, infant and young child feeding, family planning and WASH practices and knowledge; dietary diversity and minimum dietary diversity for herself and the young child; various dimensions of empowerment; and access to health and nutrition services. The grandmother questionnaire included questions on her knowledge and perceptions related to maternal and child health and nutrition, empowerment, and access to nutrition and health-related services.

| Women | Men/household head | Grandmothers |
| --- | --- | --- |
| 1.Child health and nutrition practices | 1. Demographic information | 1. Integrated nutrition knowledge and exposure |
| 1. Child health and childcare | A. HH roster | 2. Dietary recall |
| 1. Child dietary recall | B. Background information of respondents | 3. Self-efficacy |
| 1. IYCF practice | 2. HH economics | 4. *Suaahara* exposure |
| 2. Maternal health and nutrition | A. Socioeconomic status |  |
| 1. General health seeking practices | B. Economic events |  |
| 1. Antenatal care | C. Assistance |  |
| 1. Delivery and postnatal care | 3. Food security and diets |  |
| 3. Maternal dietary recall | A. HH food security |  |
| 4. Empowerment | B. Dietary recall |  |
| A. Role in HH decision-making | 4. Land use and agricultural practices |  |
| B. Productive capital | 5. Empowerment |  |
| C. Group membership | A. Role in HH decision-making |  |
| D. Freedom of movement autonomy | B. Productive capital |  |
| E. Time allocation  F. Division of HH labor | C. Group membership |  |
| 5. Water, Sanitation and Hygiene | D. Freedom of movement autonomy |  |
| 6. Integrated nutrition knowledge and exposure | E. Time allocation |  |
| 7. Self-efficacy, gender-based violence, and psychosocial well-being | 6. Integrated nutrition knowledge and exposure |  |
| 8. *Suaahara* exposure | 7. Self-efficacy, and psychosocial well-being |  |
| 9. COVID-19 module | 8. *Suaahara* exposure |  |
|  | 9. Observations |  |

Appendix 6. Reduction in gaps by socioeconomic status for comparison and intervention arms.

|  | **Comparison** | | | | | | | | **Intervention** | | | | | | | |  |  |
| --- | --- | --- | --- | --- | --- | --- | --- | --- | --- | --- | --- | --- | --- | --- | --- | --- | --- | --- |
|  | **Baseline** | |  | **Endline** | |  |  |  | **Baseline** | |  | **Endline** | |  |  |  |  |  |
|  | **Upper SES** | **Lower SES** | **Gap** | **Upper SES** | **Lower SES** | **Gap** | **DID** | **Pvalue** | **Upper SES** | **Lower SES** | **Gap** | **Upper SES** | **Lower SES** | **Gap** | **DID** | **Pvalue** | **Diff DID** | **Pvalue** |
| Maternal minimum dietary diversity | 35.1 | 19.6 | 15.5 | 31.0 | 14.9 | 16.1 | 0.6 | 0.741 | 31.3 | 20.9 | 10.4 | 36.4 | 37.4 | -1.0 | -11.4 | 0.086 | -12.0 | 0.155 |
| Child minimum dietary diversity (6-23.9m) | 48.7 | 43.1 | 5.6 | 66.5 | 56.3 | 10.2 | 4.6 | 0.583 | 45.6 | 36.5 | 9.1 | 68.6 | 68.1 | 0.5 | -8.6 | 0.251 | -13.2 | 0.533 |
| Early initiation of breastfeeding (0-23.9m) | 51.2 | 41.5 | 9.7 | 62.3 | 72.4 | -10.1 | -19.8 | 0.084 | 35.7 | 33.3 | 2.4 | 62.6 | 77.0 | -14.4 | -16.8 | 0.008 | 3.0 | 0.466 |
| Introduction of complementary feeding (6-23.9m) | 20.9 | 16.7 | 4.2 | 24.0 | 35.3 | -11.3 | -15.5 | 0.124 | 11.0 | 15.1 | -4.1 | 28.6 | 38.6 | -10.0 | -5.9 | 0.786 | 9.6 | 0.798 |
| Minimum Meal Frequency (6-23.9m) | 77.3 | 88.8 | -11.5 | 81.7 | 80.0 | 1.7 | 13.2 | 0.300 | 68.0 | 63.5 | 4.5 | 83.3 | 84.8 | -1.5 | -6.0 | 0.388 | -19.2 | 0.567 |
| Minimum Acceptable Diet (6-23.9m) | 41.8 | 36.5 | 5.3 | 49.4 | 46.6 | 2.8 | -2.5 | 0.801 | 35.5 | 24.0 | 11.5 | 56.1 | 54.3 | 1.8 | -9.7 | 0.002 | -7.2 | 0.151 |
